# Utility of Microwave-Synthesized Silver Nano Particles as Spectrofluorimetric Sensors for the Determination of Nano Concentrations of Favipravir: Application to Dosage Forms and Spiked Human Plasma

**DOI:** 10.1007/s10895-024-03979-0

**Published:** 2024-10-19

**Authors:** Mona H. Abo Zaid, Nahed El-Enany, Aziza E. Mostafa, Ghada M. Hadad, Fathalla Belal

**Affiliations:** 1 Pharmaceutical Chemistry Department, Faculty of Pharmacy, Delta University for Science and Technology, Gamasa, 35712 Egypt; 2 Pharmaceutical Analytical Chemistry Department, Faculty of Pharmacy, Suez Canal University, Ismailia, 41522 Egypt; 3 Pharmaceutical Analytical Chemistry Department, Faculty of Pharmacy, Mansoura University, Mansoura, 35516 Egypt; 4 Pharmaceutical Chemistry Department, Faculty of Pharmacy, New Mansoura University, New Mansoura, 7723730 Egypt

**Keywords:** Silver-nanoparticles, Microwave synthesis, Chitosan, Favipiravir

## Abstract

A simple and facile microwave-assisted method was developed for the synthesis of highly fluorescent silver-nanoparticles (Ag-NPs). The synthesis of silver-nanoparticles depends on a redox reaction between silver nitrate and ascorbic acid using chitosan as a stabilizing agent. The produced Ag-NPs were characterized using Zeta potential and transmission electron microscope micrograph where they are spherical in shape with smooth surface morphology and size of 26.81 ± 2.2 nm. Favipiravir (FAV) was found to cause an obvious enhancement in the fluorescence of Ag-NPs; hence, they were used for its spectrofluorimetric estimation. The fluorescence intensity was measured at 430 nm after excitation at 360 nm. Under optimum conditions, a good linear relationship was accomplished between the FAV concentration and the fluorescence intensity in a range of (5.0–200.0) ng/mL with a limit of detection of 1.59 ng/mL. The method was successfully applied for the assay of the drug in its commercial tablets and spiked human plasma samples, and the results obtained were satisfactory.

## Introduction

During last decade, the development of nanotechnology is predicted to be the establishment of a technological evolutionary of this modern era. Recently, nanotechnology is one of the most active subjects of substantial research in modern material sciences and hence metal nanoparticles have a great scientific interest because of their unique optoelectronic and physicochemical properties with applications in diverse areas, such as electronics, catalysis, drug delivery, or sensing [1]. Nanotechnology also involves synthesis of nanoparticles of size ranging from 1 to 100 nm. The wide applicability of nanoparticles is due to their size, structure, optical, chemical and physical properties [2, 3]. Metal nanoparticles have been used in a wide-ranging application in various fields. Technologies based on nanoscale materials have been exploited in a variety of fields from chemistry to medicine [4]. Recently, silver nanoparticles (Ag-NPs) have been investigated extensively due to their superior physical, chemical, and biological characteristics, and their superiority stems mainly from the size, shape, composition, crystallinity, and structure of Ag-NPs compared to their bulk forms [5]. Silver-nanoparticles (Ag-NPs) have wide-range of antimicrobial activities besides their enormous uses in many fields especially those related to drug delivery and analysis [6, 7]. In the field of drug analysis, Ag-NPs are utilized to develop many sensitive and green analytical methodologies for the determination of drugs at the lowest cost due to their primary dependence on the water as a cheap and green solvent [8–12]. Furthermore, the Ag-NPs-enhanced fluorescence technique is a promising trend in today’s spectrofluorimetric experiments. Fluorescence enhancement is effectively used to improve the techniques sensitivity and detection limits to nano-levels to be more convenient for quantitative drug analysis in miscellaneous matrices at ultra-trace levels [10, 11].

**Fig. 1:**
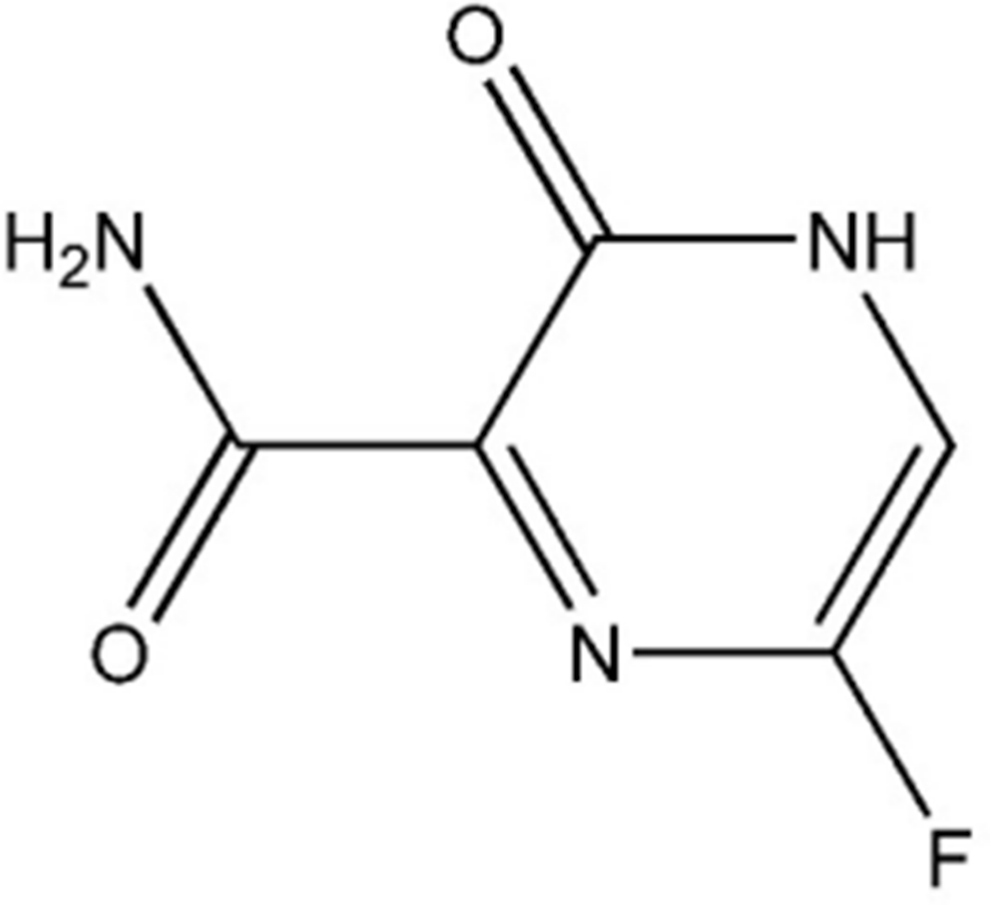
Structural formula of Favipiravir

The aim of this work is to synthesize highly fluorescent Ag-NPs using green synthesis method to be used as nano-sensors for spectrofluorimetric determination of favipiravir. Favipiravir, FAV (Fig. 1) is a potent antiviral agent that inhibits viral RNA-polymerase. It has pKa value of 5.1 and log P values 0.25 & 0.49 [13]. FAV was approved first in Japan in 2014 for treatment of Influenza. It’s a pro-drug which must be activated first in-vivo by a phosphoribosylation step into the active form which inhibits viral protein synthesis. Several clinical trials were made to test the efficacy of FAV in Corona virus infections and declared to enhance viral clearance and improve chest CT [14–16]. The side effects associated with the use of FAV were found to be mild and manageable. Recently, FAV was approved in several countries for the treatment of COVID-19 infections, including Italy, Japan, Russia, Egypt, India, KSA, UAE and Turkey [17].

Literature review revealed few methods for determination of FAV. The reported analytical approaches for assay of FAV in biological fluids and dosage forms including spectrophotometry, LC-MS, HPTLC, RP-HPLC and spectrofluorimetric were reviewed by Pruthvishree and Zaranappa [18]. Most of the reported methods depended on chromatographic techniques [19, 20]. Those techniques have several drawbacks, including expensive instrumentation, increased volumes of organic solvents and the necessity for highly qualified individuals. Also, chromatographic methods are time-consuming and need complex sample pre-treatment processes. The reported spectrophotometric methods are less sensitive and specific than the proposed one [21, 22]. Similarly, the reported spectrofluorimetric methods are less sensitive than the proposed method [19, 23] as summarized in Table 1.

**Table 1:** Comparison between the proposed method and the reported methods

Parameter	The proposed method	[19]	[20]	[21]	[22]	[23]
Technique	Spectroscopic	Chromatographic and spectroscopic	chromatographic	Spectroscopic	spectroscopic	spectroscopic
Linearity range	(5.0-200.0 ng/mL)	(20.0–350 ng/mL) for spectroscopic method & 10.0–100 µg/mL for chromatographic method	0.10 to 100.0 µg/mL	1.0–20.0 µg/mL	0.18 and 0.55 µg/mL	10.0- 100 .0ng/mL
Organic solvent	Free	Free in spectroscopic method	50 mM phosphate buffer (pH = 2.5) and acetonitrile in a ratio of 60: 40, v/v.	Ethanol	Methanol, ethanol, and water in the ratio of 25:35:40 (v/v/v)	Free

The present work describes a simple, sensitive, low-cost and rapid spectrofluorimetric method for the assay of FAV based on the use of Ag-NPs. The method of synthesis of Ag-NPs is eco-friendly and requires fast and simple procedures. Ten minutes time interval was enough to ensure the complete production of Ag-NPs.

## Experimental

### Reagents and Materials

HPLC grade methanol was purchased from Fisher Chemicals (Geel, Belgium). Boric acid and Ortho-phosphoric acid (85%) were purchased from Sigma-Aldrich(Switzerland).Glacial acetic acid 99% was purchased from Alfa Chemical Group(Cairo, Egypt). Britton-Robinson buffer (BRb) was prepared by mixing 0.04 M solution of each of a cetic acid, boric acid and phosphoric acid, the pH was adjusted over the range of 2.1 to 12 using 0.2 M NaOH. Silver nitrate was purchased from Merck, Germany. Solution of 10 mM was prepared by dissolving 0.17 g silver nitrate in 100 mL water. Chitosan (M. Wt. 100,000-300,000) was purchased from Acros Organics, Geel, Belgium. Solution of 3 g% was prepared by dissolving 3 g chitosan in 100 mL 1% acetic acid. Ascorbic acid was purchased from Sigma-Aldrich, Switzerland. Solution of 10 g% was prepared by dissolving 10 g ascorbic acid in 100 mL water and used throughout the work. Favipiravir pure sample (99.9%, certified purity) was kindly supplied by NODCAR, Cairo, Egypt. Avipiravir Tablets: 200 mg/tablet (LOT No. 2110656), product of EVA Group Pharmaceutical Company (Cairo, Egypt) and were obtained from local Pharmacy. Deionized water was used all over the study. Human plasma samples were generously provided by Mansoura University Hospital, Mansoura, Egypt, and were kept frozen at − 80 °C until use after gentle thawing.

### Apparatus

Domestic LG microwave (Power output of 900 W and frequency of 2,450 MHz) was utilized for the synthesis of Ag-NPs. Shimadzu RF-6000 Series Spectrofluorometer with 150 W Xenon lamp was utilized all over the study for the spectrofluorimetric measurements. JEM-2100 high resolution transmission electron microscope (HRTEM) from JEOL (Tokyo, Japan) was used to identify particle size of Ag-NPs. Zeta potential analyzer (Malvern Zetasize Nano-zs90) was used to measure zeta potential. pH- Meter, Jenway3510(UK), Centrifuge, model Sigma 2-16P(Germany), and Vortex mixer, ZX3 (Velp. Scientific, Italy) were used throughout the experiment.

### Standard Solutions

Stock solution (1000.0 µg/mL) of FAV was prepared by dissolving 10.0 mg in methanol in 10- mL volumetric flask. Working solutions were obtained by appropriate serial dilutions of the stock solution.

### Synthesis of Ag-NPs

100 mL Aliquot of silver nitrate solution was mixed with 100 mL of chitosan solution and 10 mL of ascorbic acid solution. The reduction reaction was carried out by heating in a microwave oven for 10 min. The produced colloidal suspension was diluted with deionized water and centrifuged for 10 min at 6000 rpm. The produced yellow colored solution was filtered through 0.22 μm syringe filters. Finally, the volume of Ag-NPs was adjusted using deionized water to get a final volume of 100 mL to prepare the stock solution [24].

## General Procedure

### Calibration Plot

**Fig. 2:**
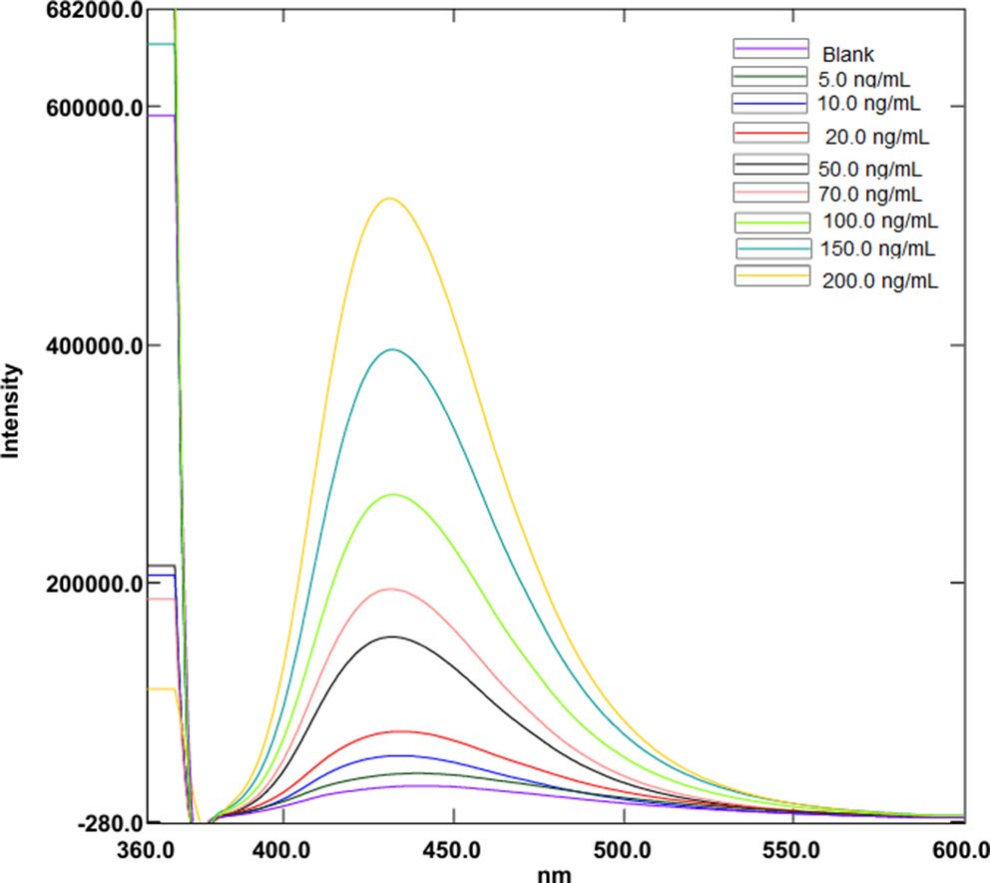
Fluorescence emission spectra of Ag-NPs in aqueous solution upon addition of various concentrations of FAV (from bottom to top: 0 ng/mL, 5.0ng/mL, 10.0ng/mL, 20.0ng/mL, 50.0ng/mL, 70.0ng/mL, 100.0ng/mL, 150.0ng/mL, 200.0ng/mL)

Aliquots of 1.0 mL of Ag-NPs were accurately transferred into a series of 10-mL volumetric flasks. 1 mL Aliquots of BRb of pH 11 were then added followed by increasing volumes of the working standard solution of FAV (1.0 µg/mL). The volume was made up to the mark with deionized water. The values of fluorescence enhancement (ΔF) were measured at 430 nm after excitation at 360 nm (Fig. 2). The plot of ΔF against final drug concentration in (ng/mL) was constructed. Subsequently, the linear regression equation was derived.

## Applications

### Application to Commercial Tablets

Ten of avipiravir tablets were weighed individually then transferred to a mortar and pulverized. An amount of the powder equivalent to 10.0 mg of FAV was accurately transferred into a 10-mL volumetric flask then 5 mL of methanol was added. Sonication was carried out for ten minutes then the volume was made up to the mark with methanol to give a solution having concentration of 1000 µg/mL, then the solution was filtered. Dilution with methanol was performed to get a solution of concentration (1.0 µg/mL). The described procedure under Section (**Calibration plot)** was then applied. The nominal contents of the tablets were calculated adopting the regression equation.

### Application to Spiked Human Plasma

1.0 mL Aliquots of human plasma samples were transferred into a series of 15-mL centrifuge tubes followed by aliquots of FAV working solution were added, then vortex-mixed for thirty seconds. Plasma protein precipitation was performed via addition of up to 5 mL of acetonitrile. The tubes were centrifuged at 3600 rpm for twenty minutes. Aliquots of 1.0 mL of the clear supernatant were transferred into 10-mL measuring flasks and the procedure under Section (**Calibration plot**) was then applied. The regression equation was used to calculate the percentage recoveries.

## Results and Discussion

### Characterization of Ag-NPs

**Fig. 3:**
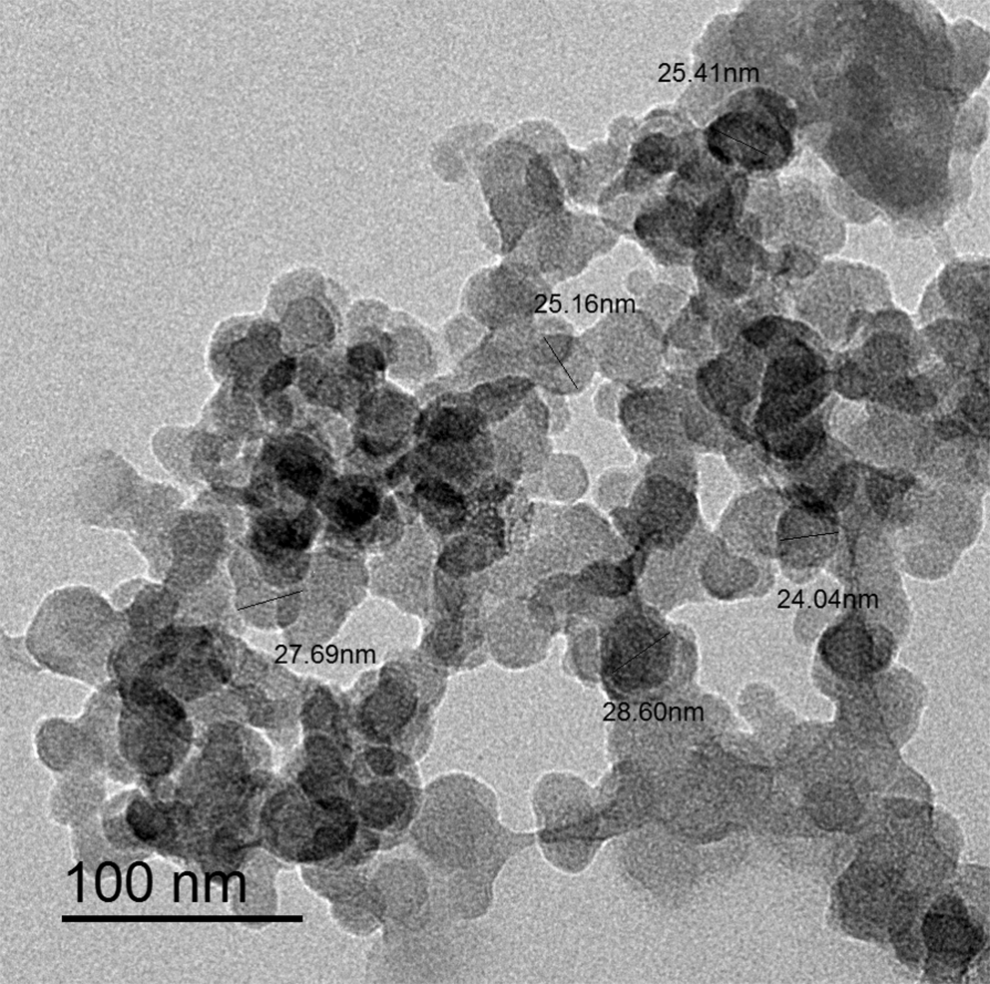
Typical HRTEM images of the prepared Ag-NPs

**Fig. 4:**
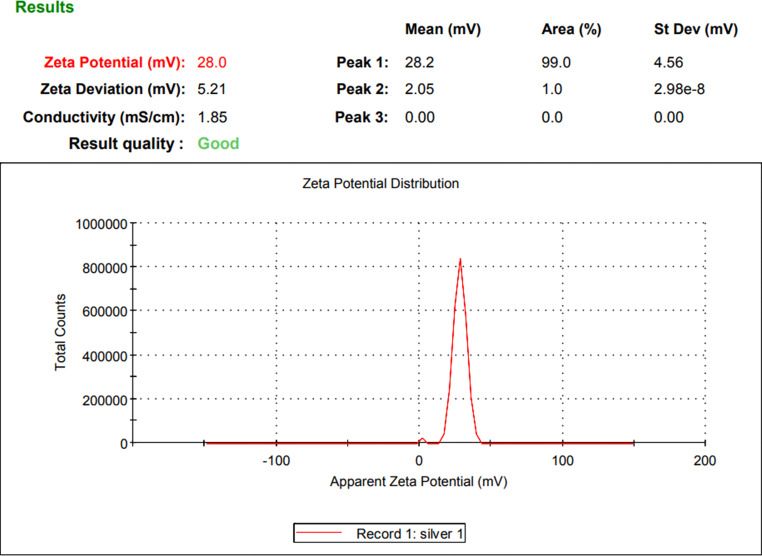
Zeta potential for the prepared Ag-NPs

Fluorescent Ag-NPs were prepared by mixing silver nitrate solution (10mM) with chitosan solution and ascorbic acid solution. The reduction reaction was carried out by heating in a microwave oven for 10 min. Different concentrations of silver nitrate were tried (10, 30, 50 mM) and it was found that, 10mM gave the smallest particle size in the range of 24.04–28.60 nm with average size of 26.81 nm (Fig. 3). Also, different concentrations of chitosan were tried (1, 2, 3% W/V) and it was found that 3% (W/V) solution gave the highest Zeta potential indicating the highest stability (Fig. 4) because chitosan act as stabilizing agent. Different heating time intervals were tried from 4 to 12 min. It was found that from 4 to 8 min heating time is not enough to produce Ag-NPs. After heating for 8 min, Ag-NPs were produced with but higher particle size. Heating for 10 min gave the smallest particle size. While after 12 min heating, the produced Ag-NPs showed particles aggregations indicated by the grey color resulting after dilution with deionized water. So, 10 min was chosen as the optimum heating time.

### Quantum Yield

In the current study, the prepared Ag-NPs were found to have high quantum yield (0.32). The single point method was adopted to calculate the quantum yield following the formula in Eq. 1 [25].

1 $$\phi_{x}\hspace{0.17em}=\hspace{0.17em}\phi_{st}\:\times\:\:({F}_{x}/{F}_{st})\:\times\:\:(\eta_{x}/\eta_{st})\:\times\:\:({A}_{st}/{A}_{x})$$

where:

Φ represents the quantum yield,

(st) and (x) subscripts refer to the standard solutions and the unknown sample.

F is the integrated measured intensity of emission,

A is the absorbance and η represents the refractive index of the solvent.

In aqueous solutions, η_x_/η_st_ is equal to 1. Quinine sulfate in 0.1 M H_2_SO_4_ was used as the standard fluorescent substance (QY: 0.54).

## Method Development and Optimization

### Effect of pH

**Fig. 5:**
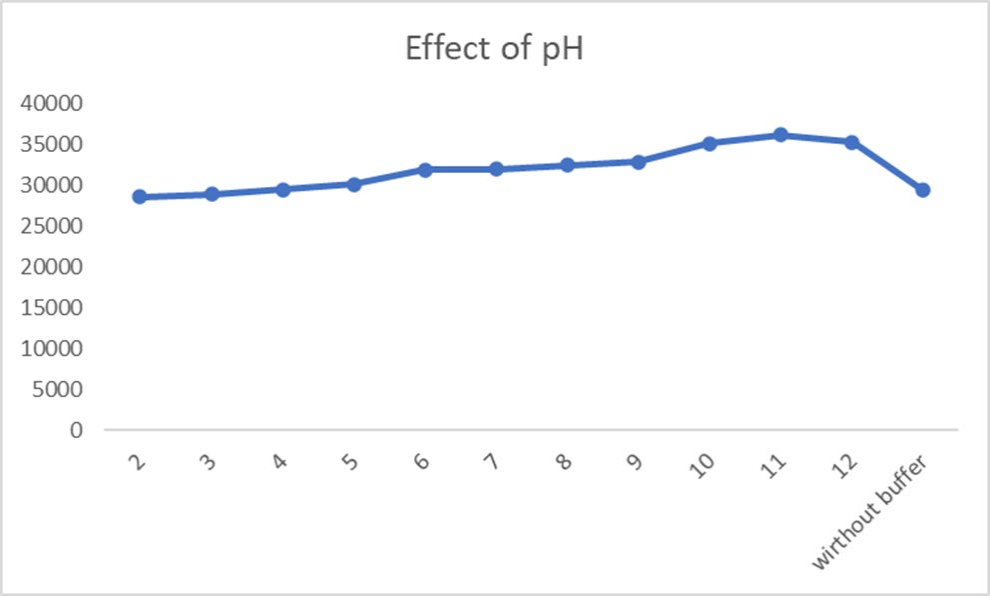
Effect of pH on the RFI of FAV

The influence of pH on the fluorescence intensity enhancement by FAV was investigated using BRb over the pH range of 2.1–12. The highest relative fluorescence intensity (RFI) value was achieved at pH 11 (Fig. 5).

### Effect of Buffer Volume

To study the influence of volume of buffer, increasing volumes of BRb were used. It was observed that the maximum enhancement effect on the fluorescence of Ag-NPs was achieved upon adding 1 mL of BRb buffer of pH 11 while using higher or lower volumes causes lower enhancement.

### Effect of Incubation Time

After the addition of FAV solution to Ag-NPs, the fluorescence emission spectra were recorded at several time intervals starting from 1 to 30 min. The interaction was found to be instantaneous and 1 min was enough to be complete. The fluorescence intensities were stable for at least 30 min.

### Method Validation

The ICHQ2(R1) guidelines were used to confirm the validity of the suggested method [26]. The plot of concentrations of the drug in ng/mL versus the relative fluorescence intensity was constructed to evaluate the linearity of the suggested method. The method was found to be linear over the range of 5.0 to 200.0 ng/mL. The linear regression is represented in Eq. 2:

2 $$RFI= 2464.94C-701.63\quad\quad(R^2=0.9999)$$

where RFI is the relative fluorescence intensity, C is FAV concentration in ng/mL.

The method was confirmed to be sufficiently sensitive as values of limits of detection (LOD) and quantitation (LOQ) are quite low, the results are summarized in Table 2. The mean % recoveries of raw material of FAV were calculated to confirm the accuracy of the suggested method. The comparison method relies on measuring the fluorescence intensity of FAV at 436 nm after excitation at 323 nm using water as diluting solvent [19]. The Variance ratio F-test and Student t-test were used to compare the results obtained from the comparison and suggested methods as illustrated in Table 3. There was no significant difference between the two methods regarding accuracy and precision.

**Table 2:** Analytical performance data for the proposed method

Parameter	FAV
Linearity range (ng/mL)	5.0–200.0
Limit of detection (LOD)	1.59
Limit of quantitation (LOQ)	4.85
Correlation coefficient	0.9999
S.D. of residuals (S_y/x_)	2219.45
S.D. of intercept (S_a_)	1195.11
S.D. of slope (S_b_)	11.92

**Table 3:** Assay results for the determination of the studied drug in raw material by the proposed and comparison methods

Parameter	Proposed method			Comparison method ^[19]^
	Concentration taken ^a^ (ng/mL)	Concentration found (ng/mL)	% Found	% Found
	5.0 10.0 20.0 50.0 70.0 100.0 150.0 200.0	5.08 10.15 19.69 50.69 68.85 101.14 148.84 200.55	101.68 101.55 98.43 101.38 98.36 101.14 99.23 100.27	101.57 99.31 100.15
X̄± SD			100.26 ± 1.4	100.34 ± 1.14
*t-*test			0.08 (2.26)^b^	
*F*-test			1.5(19.35)^b^	

^a^Each result was the average of three separate determinations

^b^ The figures between brackets were the tabulated *t* and *F* values at *P* = 0.05 [27]

The precision of the method was assessed by exploring the intraday and inter-day precisions. The %RSD values were calculated using three replicates of three different concentrations on the same day and on three successive days. The proposed method showed low values of % RSD, lower than 2% which are acceptable (Table 4).

**Table 4:** Intraday and inter-day precision data for the determination of the studied drug by the proposed method

Conc. taken in ng/mL	Intraday ^a^			Inter-day ^b^		
	Conc. found ± S.D. (ng/mL)	%RSD	%Error	Conc. found ± S.D. (ng/mL)	%RSD	%Error
20.0	20 ± 0.34	0.34	0.19	20 ± 0.72	0.73	0.42
50.0	50 ± 0.30	0.29	0.17	50 ± 1.16	1.16	0.67
70.0	70 ± 0.38	0.38	0.22	70 ± 0.76	0.76	0.44

Each result is the average of three separate determinations

^a^ Within the day

^b^ Three consecutive days

Altering the volume of buffer by ± 2mL and the pH by ± 0.2 caused no significant changes in the results of the study. Those small and minor changes may occur throughout routine work. Consequently, the method proved to be robust.

The selectivity of the suggested method was confirmed by calculating the % recoveries of FAV in its commercial tablets as shown in Table 5. From the obtained results, it was clear that there was no interference from common tablet excipients with the results of the method. Accordingly, the method could be applied for the determination of FAV in its commercial tablets.

**Table 5:** Assay results for determination of FAV in its dosage forms by the proposed method

Preparation	Proposed method			Comparison method [19]
	Conc.taken^a^ (ng/mL)	Conc.found (ng/mL)	% Recovery	
Avipiravir 200 mg	20.0	19.57	97.89	101.67
	40.0	40.63	101.58	97.50
	80.0	79.79	99.74	101.33
‾x			99.74	100.17
± SD			± 1.84	± 2.32
% RSD			1.85	
% Error			1.06	
t-test			0.25(2.77)^b^	
F-test			1.58(19)^b^	

^a^ Each result was the average of three separate determinations

^b^ The figures between brackets were the tabulated *t* and *F* values at *P* = 0.05 [27]

a- Tablets contain 200 mg FAV

### Applications

#### Drug Analysis in its Commercial Tablets

FAV was effectively assayed in its commercial tablets by the proposed method. There was good agreement between the results obtained by the suggested method and those given by the comparison method as shown in Table 5. The Student t-test and Variance ratio F-test were used to statistically interpret data obtained by both methods [27]. There was no significant difference between the tabulated and calculated values regards accuracy and precision.

#### Analysis of Spiked Human Plasma

**Fig. 6:**
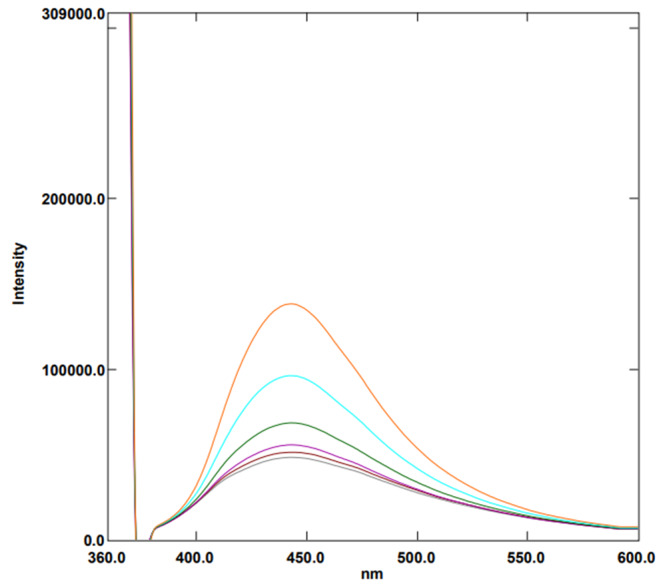
Fluorescence emission spectra of Ag-NPs under optimum conditions upon spiking of various concentrations of FAV to human plasma

After oral dosing of 200 mg FAV tablets, the peak plasma concentration was reported to be 7896 ± 2505 ng/mL [28], which lies above the working range of the proposed method, therefore, it could be used in the assay of the drug in plasma samples. The procedure described under Section (**Application to Spiked Human Plasma**) was followed after preparing the samples to obtain % recoveries for each sample. The method proved to be suitable to assay FAV in the studied matrix. The range of the average recovery values was from 93.41 to 107.13% as shown in Table 6; Fig. 6.

**Table 6:** Application of the proposed spectrofluorimetric method for determination of FAV in spiked human plasma

Drug	Conc. added (ng/mL)	Conc. found (ng/mL)	%Recoveries
FAV	10.0	9.96	99.65
	20.0	18.82	94.11
	50.0	46.71	93.41
	100.0	107.13	107.13
	200.0	197.38	98.69
x̄±SD	98.59 ± 5.49		

## Conclusion

Fluorescent nano-sensors (Ag-NPs) were successfully produced by the aid of microwave heating. The water-soluble Ag-NPs were synthesized using simple procedure. The size of Ag-NPs was in the range of 24.04 nm to 28.60 nm with an average diameter of 26.81 nm. They are stable and emitting fluorescence with quantum yield of 0.32. The produced nano-sensors were utilized to estimate FAV in its commercial tablets as well as spiked human plasma samples. The suggested method has the privilege of being highly sensitive, simple and low cost.

## Data Availability

No datasets were generated or analysed during the current study.
